# Haemodynamic goal-directed therapy and postoperative infections: earlier is better. a systematic review and meta-analysis

**DOI:** 10.1186/cc10284

**Published:** 2011-06-24

**Authors:** Lidia Dalfino, Maria T Giglio, Filomena Puntillo, Massimo Marucci, Nicola Brienza

**Affiliations:** 1 Anesthesia and Intensive Care Unit, Department of Emergency and Organ Transplantation, University of Bari, Piazza G. Cesare 11, I-70124 Bari, Italy

## Abstract

**Introduction:**

Infectious complications are the main causes of postoperative morbidity. The early timing of their promoting factors is the rationale for perioperative strategies attempting to reduce them. Our aim was to determine the effects of perioperative haemodynamic goal-directed therapy on postoperative infection rates.

**Methods:**

We performed a systematic review and meta-analysis. MEDLINE, EMBASE, The Cochrane Library and the DARE databases were searched up to March 2011. Randomised, controlled trials of major surgery in adult patients managed with perioperative goal-directed therapy or according to routine haemodynamic practice were included. Primary outcome measure was specific type of infection.

**Results:**

Twenty-six randomised, controlled trials with a combined total of 4,188 participants met our inclusion criteria. Perioperative goal-directed therapy significantly reduced surgical site infections (pooled OR 0.58, 95% CI 0.46 to 0.74; *P *< 0.0001), pneumonia (pooled OR 0.71, 95% CI 0.55 to 0.92; *P *= 0.009), and urinary tract infections (pooled OR 0.44, 95% CI 0.22 to 0.84; *P *= 0.02). A significant benefit was found regarding total infectious episodes (OR 0.40, 95% CI 0.28 to 0.58; *P *< 0.00001).

**Conclusions:**

Flow-directed haemodynamic therapy designed to optimise oxygen delivery protects surgical patients against postoperative hospital-acquired infections and must be strongly encouraged, particularly in the high-risk surgical population.

## Introduction

In major surgical patients, infectious complications are the main cause of morbidity [1,2]. Other than strict asepsis, antibiotic prophylaxis, avoidance of glucose imbalance, and normothermia [3], no strategy has been demonstrated to be effective for their prevention. Even though postoperative infections are typically not detected until a few days after surgery, their promoting factors occur within the first few hours following surgical insult.

Goal-directed therapy (GDT) is a haemodynamic treatment based on titration of fluid and inotropic drugs infused to physiologic flow-related end points [4]. This regimen was originally applied in surgical patients with the aim of reaching normal or supranormal values of cardiac output and oxygen delivery (DO_2_) to manage the perioperative increase in oxygen demand and to prevent organ failure [5]. When performed perioperatively in high-risk surgical patients, GDT decreases mortality [6] and postoperative renal and gastrointestinal complications [7,8]. However, no study on perioperative GDT has specifically assessed postoperative infections as the main primary outcome measure.

Therefore, we performed a systematic review including a meta-analysis about the effects of perioperative GDT on postoperative infections. We reviewed randomised, controlled trials (RCTs) to assess the efficacy of GDT compared with standard haemodynamic management of postoperative infections in adult surgical patients.

## Materials and methods

### Eligibility criteria

Studies were searched according to the following eligibility criteria [9]:

1. Types of participants: Adult patients (ages 18 years and older) undergoing major surgery were considered. Studies involving mixed populations of critically ill, nonsurgical patients, or postoperative patients with sepsis or organ failure were excluded.

2. Types of interventions: GDT was defined as perioperative monitoring and manipulation of haemodynamic parameters to reach normal or supranormal values by fluid infusion alone or in combination with inotropic therapy within eight hours after surgery. Studies including late haemodynamic optimisation treatment were excluded.

3. Types of comparisons: Trials comparing the beneficial and harmful effects of GDT and standard haemodynamic therapy were considered. We excluded RCTs with no description of or no difference in optimisation strategies between groups, as well as RCTs in which therapy was titrated to the same goal in both groups or was not titrated to predefined end points.

4. Types of outcome measures: The primary outcome measures were surgical site infections (SSIs), either incisional and organ or space; pneumonia; catheter-related bloodstream infections (CRBSIs), and urinary tract infections (UTIs). The secondary outcome measure was the number of overall postoperative infectious episodes.

5. Types of studies: RCTs on perioperative GDT in surgical patients were included. No language, publication date, or publication status restrictions were imposed.

### Information sources

Different search strategies (last update March 2011) were performed to retrieve relevant studies by using the MEDLINE, The Cochrane Library and EMBASE databases. No date restriction was applied for searching the MEDLINE and The Cochrane Library databases, while the search was limited to 2007 to 2011 for searching of the EMBASE database [10]. Additional RCTs were searched in The Cochrane Library and the Database of Abstracts of Reviews of Effects (DARE) databases and in the reference lists of previously published reviews and retrieved articles. Other data sources were hand-searched in the annual proceedings (2003 to 2010) of the Society of Critical Care Medicine, the European Society of Intensive Care Medicine, the Society of Cardiovascular Anesthesiologists, the Royal College of Anaesthetists and the American Society of Anesthesiologists.

### Search terms

Trials selection was performed by using the following search terms: randomized controlled trial, controlled clinical trial, surgery, goal directed, goal oriented, goal target, cardiac output, cardiac index, DO_2_, oxygen consumption, cardiac volume, stroke volume, fluid therapy, fluid loading, fluid administration, optimisation, optimization, sopranormal, supranormal.

The search strategies used for the MEDLINE, The Cochrane Library and EMBASE databases are reported in Additional file 1.

### Study selection

Two investigators (MM and LD) first examined each title and abstract to exclude clearly irrelevant studies and to identify potentially relevant articles. Other two investigators (MG and FP) independently determined the eligibility of full-text articles retrieved. The names of the authors, institutions, and the journals in which the articles were published, as well as the RCT's results, were unknown to the two investigators at that time.

### Data collection process

Data were independently collected by two investigators (MG and NB), with any discrepancy being resolved by reinspection of the original article. To avoid transcription errors, the data were input into statistical software and rechecked by different investigators (FP and LD).

### RCT data gathered

Data abstraction included patient characteristics (age and sex) and risk factors (based on Physiologic and Operative Severity Score for the Enumeration of Mortality and Morbidity - POSSUM score [11], American Society of Anesthesiologists Physical Status classification, age > 60 years and preoperative morbidity), type of surgery (that is, elective or emergent, abdominal, thoracic, vascular and so forth), type of haemodynamic GDT (monitoring tools, haemodynamic end points and therapeutic interventions), incidence (number of infected patients and number of infectious episodes) and definition of postoperative infections.

### Risk of bias in individual studies

The Scottish Intercollegiate Guidelines Network (SIGN) checklist for RCTs was used to evaluate the methodological quality of the RCTs. The SIGN checklist was independently filled in by two investigators (MG and MM) and whenever their results differed, the study was further assessed to reach consensus. In Table 1, a double-plus sign (++) denotes studies very unlikely to have bias, a single plus sign (+) indicates studies in which bias is unlikely and a minus sign (-) denotes studies with a high risk of bias [12]. A double-plus sign was assigned to studies that adequately fit all the criteria of randomisation, concealment, blinding, intention-to-treat analysis and predefined outcomes. A single plus sign was given to studies meeting only four of the five criteria. The adequacy of these five criteria is strongly associated with bias reduction [13,14]. Regarding blinding, those studies in which the outcome was explicitly predefined, the outcome assessment was blinded, or both, were considered adequately masked [15]. For every site of infection, a quality-sensitive analysis was performed, including studies with a low risk of bias (SIGN evaluation ++ or +) and those with a high risk of bias (SIGN evaluation -), and a subgroup analysis was planned that included only trials that enrolled high-risk patients. Moreover, an outcome-level assessment (sensitivity analysis) was performed, including studies providing definitions consistent with the Centers for Disease Control and Prevention (CDC) criteria [16].

**Table 1:** Quality assessment, sample characteristics and intervention details of the included studies^a^

Study	SIGN score	SIGN comment	Risk definition	Type of surgery	Goal-directed therapy (tools and goals)	Modality of optimisation
Bender *et al*. 1997 [21], USA	-	Randomisation and concealment not clear, not blinded		Elective aortic and vascular	PAC: CI ≥ 2.8 L/minute/m^2^, 8 ≤ PCWP ≤ 14 mmHg, SVR ≤1,100 dyn/second/cm^5^	Fluids and inotropes
Benes *et al*. 2010 [22], Europe	++		High risk	Major abdominal	Vigileo monitor/FloTrac sensor: SVV < 10%	Fluids and inotropes
Bishop *et al*. 1995 [23], USA	-	Randomisation not adequate, concealment not clear, not blinded	High risk	Emergent trauma	PAC: CI ≥ 4.5 L/minute/m^2^, DO_2 _≥ 670 mL/minute/m^2^, VO_2 _≥ 166 mL/minute/m^2^	Fluids and inotropes
Boyd *et al*. 1993 [24], Europe	-	Randomisation and concealment not clear	High risk	Emergent or elective major abdominal or vascular	PAC: DO_2 _> 600 mL/minute/m^2^	Fluids and inotropes
Chytra *et al*. 2007 [25], Europe	+	Randomisation not clear	High risk	Emergent trauma	Oesophageal Doppler: SV optimisation with FTc > 0.35 seconds	Fluids (noradrenaline intraoperatively)
Fleming *et al*. 1992 [26], USA	-	Randomisation not adequate, not blinded, concealment not described	High risk	Emergent trauma	PAC: CI ≥ 4.5 L/minute/m^2^, DO_2 _≥ 670 mL/minute/m^2^, VO_2 _≥ 166 mL/minute/m	Fluids and inotropes
Forget *et al*. 2010 [27], Europe	++		High risk	Major abdominal	Masimo SET pulse oximeter: PVI > 13%	Fluids
Gan *et al*. 2002 [28], USA	++			Elective general, urologic, gynaecologic	Oesophageal Doppler: SV optimisation with FTc between 0.35 and 0.4 seconds	Fluids
Jhanji *et al*. 2010 [29], Europe	++		High risk	Major elective abdominal	LiDCO Cardiac Sensor System: SV > 10%	Fluids
Lobo *et al*. 2000 [30], Brazil	+	Randomisation not clear	High risk	Elective major abdominal or vascular	PAC: DO_2 _> 600 mL/minute/m^2^	Fluids
Lopes *et al*. 2007 [31], Brazil	+	Randomisation not clear	High risk	Elective abdominal	Radial artery line: ΔPP ≤ 10%	Fluids
Mayer *et al*. 2010 [32], Europe	+	Randomisation not clear	High risk	Major abdominal	Vigileo monitor/FloTrac sensor: CI ≥ 2.5 L/minute/m^2^	Fluids and inotropes
McKendry *et al*. 2004 [33], Europe	+	Complication not defined		Elective cardiac	Oesophageal Doppler: SI > 35 mL/m^2^	Fluids
Mythen and Webb 1995 [34], Europe	-	Randomisation not clear, flow of patients not described	High risk	Elective cardiac	Oesophageal Doppler: SV optimisation and rise in CVP < 3 mmHg	Fluids
Noblett *et al*. 2006 [35], Europe	+	Randomisation not clear		Colorectal	Oesophageal Doppler: SV optimisation with FTc between 0.35 and 0.4 seconds	Fluids (catecholamines intraoperatively)
Pearse *et al*. 2005 [36], Europe	++		High risk	Elective or emergent major general	LiDCO Cardiac Sensor System: DO_2 _> 600 mL/minute/m^2^, SV > 10%	Fluids and inotropes
Sandham *et al*. 2003 [37], Canada	++		High risk	Elective or emergent major abdominal, thoracic, vascular, or orthopaedic	PAC: CI > 3.5 and < 4.5 L/minute/m^2^, 550 < DO_2 _< 600 mL/minute/m^2^, MAP > 70 mmHg, PCWP < 18 mmHg	Fluids and inotropes
Shoemaker *et al*. 1998 [38], USA	-	Not blinded, unclear dropouts and withdrawals	High risk	Emergent or elective major abdominal (general or vascular)	PAC: CI > 4.5 L/minute/m^2^, DO_2 _> 600 mL/minute/m^2^, VO_2 _> 170 mL/minute/m^2^	Fluids and inotropes
Sinclair *et al*. 1997 [39], Europe	-	Randomisation and concealment not clear, flow of patients not described	High risk	Orthopaedic	Oesophageal Doppler: SV optimisation with FTc between 0.35 and 0.4 seconds	Fluids
Smetkin *et al*. 2009 [40], Europe	-	Randomisation not adequate, not blinded, concealment not described		Elective cardiac	PiCCO Plus monitor: ITBVI 850 to 1,000 mL/m, ScvO_2 _> 60%	Fluids and inotropes
Valentine *et al*. 1998 [41], USA	-	Randomisation not clear, not blinded		Elective aortic	PAC: CI ≥ 2.8 L/minute/m^2^, 8 ≤ PCWP ≤ 15 mmHg, SVR ≤ 1,100 dyn/second/cm^5^	Fluids and inotropes
Van der Linden *et al*. 2010 [42], Europe	++		High risk	Vascular	Vigileo monitor/FloTrac sensor: CI > 2.5 L/minute/m^2^	Fluids and inotropes
Velmahos *et al*. 2000 [43], USA	+	Not blinded	High risk	Emergent trauma	Thoracic bioimpedance: CI > 4.5 L/minute/m^2^	Fluids and inotropes
Venn *et al*. 2002 [44], Europe	++		High risk	Orthopaedic	Oesophageal Doppler: SV optimisation with FTc > 0.4 seconds	Fluids
Wakeling *et al*. 2005 [45], Europe	++			Elective major bowel	Oesophageal Doppler: SV optimisation and rise in CVP < 3 mmHg	Fluids
Wilson *et al*. 1999 [46], Europe	+	Not blinded	High risk	Elective major (abdominal, vascular, urologic)	PAC: DO_2 _> 600 mL/minute/m^2^	Fluids and inotropes

^a^CI: cardiac index; CVP: central venous pressure; DO_2_: oxygen delivery; FTc: flow time-corrected; ITBVI: intrathoracic blood volume index; LiDCO Cardiac Sensor System (Lidco Ltd., Cambridge, UK)**; **MAP: mean arterial pressure; Masimo SET pulse oximeter (Masimo Corp., Irvine, CA, USA); PAC: pulmonary artery catheter; PCWP: pulmonary capillary wedge pressure; PiCCO Plus monitor (Pulsion Medical Systems, Munich, Germany)**; **PVI: Pleth variability index; ScvO_2_: central venous oxygen saturation; SI: stroke index; SIGN: Scottish Intercollegiate Guidelines Network; SV: stroke volume; SVR: systemic vascular resistance; SVV: stroke volume variation; VO_2_: oxygen consumption. ++Studies with very unlikely bias; +studies with unlikely bias; -studies with high risk of bias. See materials and methods section for risk definitions.

### Summary measures and planned method of analysis

Meta-analytic techniques (RevMan analytical software version 5.1.1; Cochrane Collaboration, Oxford, UK) were used to combine studies using odds ratios (ORs) and 95% confidence intervals (95% CIs). A statistical difference between groups was considered to have occurred if the pooled 95% CI did not include 1 for the OR. An OR less than 1 favoured GDT compared with control group. Two-sided *P *values were calculated. A random effects model was chosen for all analyses. Statistical heterogeneity and inconsistency were assessed by using the Q-test and *I*^2 ^test, respectively [17,18]. When the *P *value of the Q-test was < 0.10 and/or the *I*^2 ^was > 40%, heterogeneity and inconsistency were considered significant [19]. When significant heterogeneity and inconsistency were found, the most heterogeneous study on the basis of the forest plot was removed and the analysis was redone [20]. In all main analyses, studies comprising more than half the pooled number of patients were removed and reanalysis was performed.

For each main outcome measure, calculation of the number of patients needed to treat (NNT with 95% CI) to prevent one patient with complications was carried out using the formula NNT = 1*/*(RRR × CER), where RRR is the summary relative risk reduction and CER is the control event rate for all trials. Statistical power with an α error of 0.05 was calculated for each main analysis, and results ≥ 80% were considered adequate.

## Results

### Study selection

**Figure 1:**
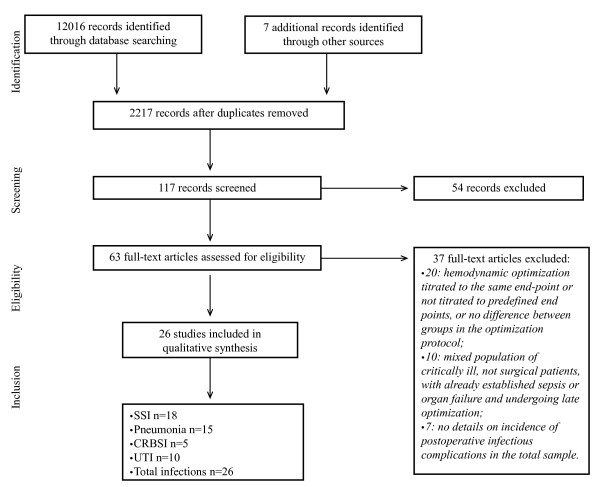
**Outline of studies selection process**. Flowchart summarising the procedure of studies selection for the meta-analysis. CRBSI: catheter-related bloodstream infection; SSI: surgical site infection; UTI: urinary tract infection.

The search strategies that we used identified 2,676 (MEDLINE), 8,545 (Cochrane Library) and 795 (EMBASE) articles. After initial screening and subsequent more detailed selection, a pool of 63 potentially relevant RCTs was identified. The eligibility process (Figure 1) excluded 37 articles, and therefore 26 RCTs [21-46] enrolling a total sample of 4,188 patients were included in our meta-analysis.

### Study characteristics

All selected articles described the results of RCTs evaluating the effects of GDT on postoperative morbidity, including infections as primary or secondary outcomes, and had a population sample of adult patients undergoing major surgical procedures. Of 26 studies, 21 studies [21-32,35-38,41-43,45,46] included abdominal surgery, 3 studies [33,34,40] involved cardiac procedures and two studies [39,44] included only orthopaedic surgery.

During the period from 1993 to 2010, 16 studies were performed in Europe [22,24,25,27,29,32-36,39,42,44-46], 7 were conducted in the United States [21,23,26,28,38,41,43], 2 were carried out in Brazil [31,32] and 1 was performed in Canada [37]. Eighteen studies reported the incidence of SSIs, fifteen reported the incidence of pneumonia, five reported the incidence of CRBSIs, and ten reported the incidence of UTIs. Nineteen studies had enrolled high-risk patients, and seventeen studies had received a SIGN evaluation of + or ++ (see Table 1 for details).

### Quantitative data synthesis

#### SSIs

**Figure 2:**
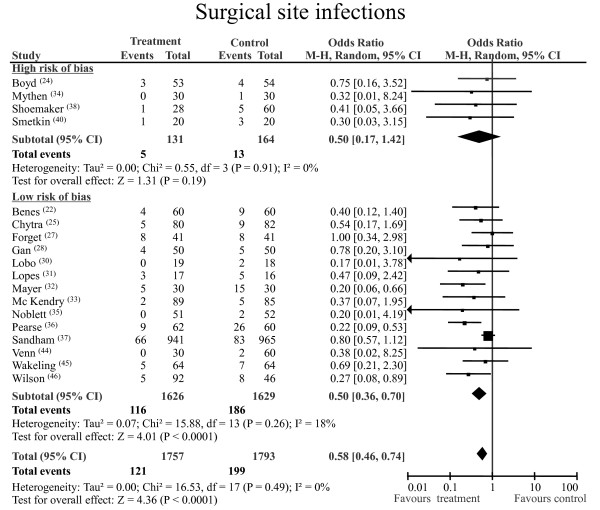
**Surgical site infections**. Rates of postoperative surgical site infections for each of the studies with OR and 95% CI data are shown. The studies were divided into two subgroups, high risk of bias or low risk of bias, according to the SIGN checklist (see materials and methods section for details). The pooled OR and 95% CI data are shown as the totals. The size of the box at the point estimate of the OR is a visual representation of the 'weighting' of the study. The diamond represents the point estimate of the pooled OR, and the length of the diamond is proportional to the 95% CI. 95% CI: 95% confidence interval; M-H: Mantel-Hentzel; OR: odds ratio; SIGN: Scottish Intercollegiate Guidelines Network.

A total of 320 episodes of SSI were registered: 199 (11%) of 1,793 occurred in patients randomised to control treatment and 121 (6.9%) of 1,757 occurred in patients randomised to the perioperative GDT group. The pooled OR was 0.58 and the pooled 95% CI was 0.46 to 0.74 (3,550 patients from 18 RCTs; statistical power 100%). The NNT was 24 (95% CI 16.4 to 42.8). No statistical heterogeneity or inconsistency was detected (Figure 2). After excluding the largest study [37], the pooled OR was 0.41 with a pooled 95% CI of 0.29 to 0.59 (*P *< 0.00001) for 1,644 patients, with no statistical heterogeneity found (*Q *statistic *P *= 0.90; *I*^2 ^= 0%). The beneficial effect of GDT in reducing SSIs was confirmed by the quality-sensitive analysis (that is, including only studies with a low risk of bias) (Figure 2).

### Pneumonia

**Figure 3:**
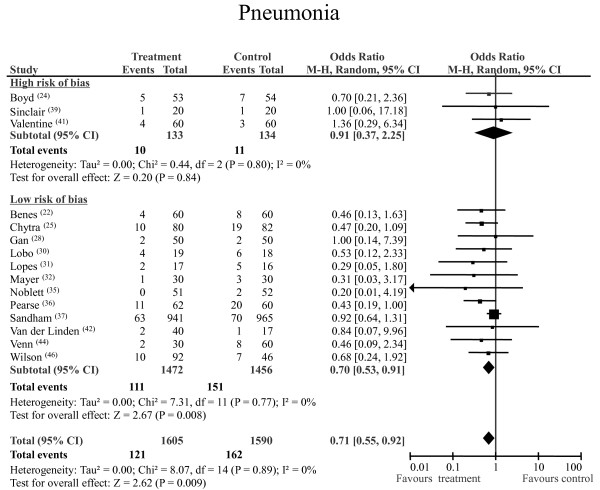
**Pneumonia**. Rates of postoperative pneumonia for each of the studies included are shown along with ORs and 95% CIs. The studies were divided into two subgroups, high risk of bias and low risk of bias, according to the SIGN checklist (see text for details). The pooled OR and 95% CI are shown as the total. The size of the box at the point estimate of the OR gives a visual representation of the 'weighting' of the study. The diamond represents the point estimate of the pooled OR and the length of the diamond is proportional to the CI.  95% CI: 95% confidence interval; OR: odds ratio; SIGN: Scottish Intercollegiate Guidelines Network.

A total of 283 episodes of postoperative pneumonia were registered: 162 (10%) of 1,590 occurred in the control group and 121 (7.5%) of 1,605 occurred in the GDT group. The pooled OR was 0.71 and the pooled 95% CI was 0.55 to 0.92 (from a total of 3,195 patients in 15 RCTs; statistical power 86%). The NNT was 34 (95% CI 20.8 to 91.6). No statistical heterogeneity or inconsistency was detected (Figure 3). Excluding the largest study [37] yielded a pooled OR of 0.54 with a pooled 95% CI of 0.38 to 0.78 (from a total of 1,289 patients, *P *= 0.001; *Q *statistic *P *= 0.99; *I*^2 ^= 0%). The quality-sensitive analysis confirmed the positive effect of GDT in studies with a low risk of bias (Figure 3).

#### CRBSIs

In five RCTs, fifty-four patients developed CRBSIs: 33 (2.7%) of 1,213 had been randomised to control group and 21 (1.7%) of 1,235 had been randomised to perioperative GDT group. GDT did not reduce the incidence of CRBSIs. The pooled OR was 0.63 (pooled 95% CI 0.31 to 1.28; statistical power 39%).

### UTIs

**Figure 4:**
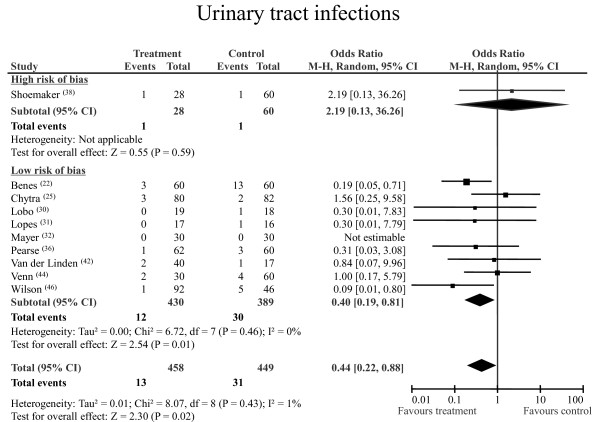
**Urinary tract infections**. Rates of postoperative urinary tract infections for each of the studies with ORs and 95% CIs. The studies were divided into two subgroups defined as high risk of bias and low risk of bias, according to the SIGN checklist (see materials and methods section for details). The pooled OR and 95% CI data are shown as the totals. The size of the box at the point estimate of the OR is a visual representation of the 'weighting' of the study. The diamond represents the point estimate of the pooled OR, and the length of the diamond is proportional to the 95% CI.  95% CI: 95% confidence interval; OR: odds ratio; SIGN: Scottish Intercollegiate Guidelines Network.

In 10 RCTs, 44 patients developed UTIs: 31 (6.9%) of 449 had been randomised to control group and 13 (2.8%) of 458 had been randomised to perioperative GDT group. GDT reduced the incidence of UTIs: the pooled OR was 0.44 (pooled 95% CI 0.22 to 0.88; statistical power 82%). The NNT was 25 (95% CI 14.6 to 78.7). No statistical heterogeneity or inconsistency was detected (Figure 4).

The quality-sensitive analysis confirmed the positive effect of GDT in studies with a low risk of bias (Figure 4). The subgroup analyses (including only high-risk patients) and the sensitivity analyses (which included studies providing definitions consistent with the CDC criteria) confirmed the main results for SSIs, pneumonia and UTIs (Table 2).

**Table 2:** Subgroup analysis for postoperative infective complications^a^

Analysis	Number of studies	References	Treatment group (episodes/total patients)	Control group (episodes/total patients)	OR (95% CI)	*P *value	*I* ^2^
SSI high-risk patients	13	[22], [24,25], [27], [30-32], [34], [36-38], [44], [46]	109/1,483	177/1,522	0.48 (0.33 to 0.70)	0.0001	21%
SSI studies providing definitions consistent with CDC criteria	8	[22], [25], [30-32], [36], [44,45]	32/362	74/390	0.37 (0.23 to 0.58)	0.0001	0%
Pneumonia high-risk patients	12	[22], [24,25], [30-32], [36,37], [39], [42], [44], [46]	115/1,444	155/1,428	0.70 (0.54 to 0.91)	0.008	0%
Pneumonia studies providing definitions consistent with CDC criteria	9	[22], [25], [28], [30-32], [36,37], [44]	99/1,289	141/1,341	0.70 (0.53 to 0.93)	0.01	0%
UTI high-risk patients	10	[22], [25], [30-32], [36], [38], [42], [44], [46]	13/458	31/449	0.44 (0.22 to 0.88)	0.02	1%
UTI studies providing definitions consistent with CDC criteria	7	[22], [25], [30-32], [36], [44]	9/298	24/326	0.45 (0.20 to 0.99)	0.05	0%

^a^95% CI: 95% confidence interval; CDC: Centers for Disease Control and Prevention; *I*^2^: inconsistency; OR: odds ratio; SSI: surgical site infection; UTI: urinary tract infection.

### All infectious episodes

**Figure 5:**
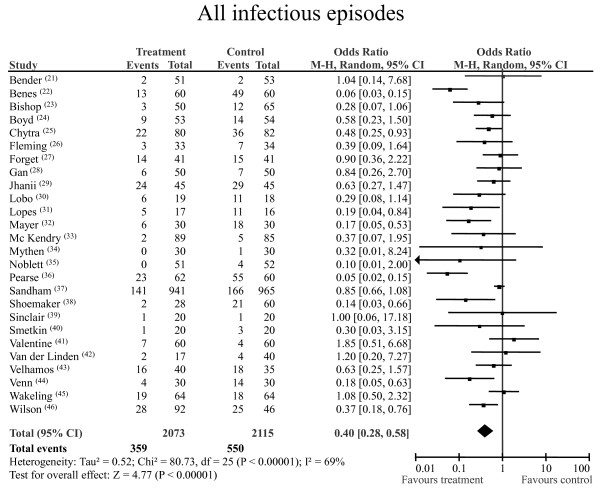
**Total postoperative infectious episodes**. Rates of total postoperative infectious episodes for each of the studies included are shown along with ORs and 95% CIs. The pooled OR and 95% CI data are shown as the totals. The size of the box at the point estimate of the OR is a visual representation of the 'weighting' of the study. The diamond represents the point estimate of the pooled OR, and the length of the diamond is proportional to the 95% CI. 95% CI: 95% confidence interval; OR: odds ratio.

In the 26 RCTs, 909 infectious events were reported: 550 (26%) in the 2,115 patients randomised to control group and 359 (17.3%) in the 2,073 patients randomised to perioperative GDT group. The pooled OR for the development of infection was 0.40 (pooled 95% CI 0.28 to 0.58; statistical power 100%). The NNT was 12 (95% CI 9.0 to 16.1). Statistically significant heterogeneity and inconsistency were detected (Figure 5). After excluding the largest study [37], leaving a combined sample of 2,282 patients, the pooled OR was 0.38 and the pooled 95% CI was 0.26 to 0.56 (*P *< 0.0001), but statistically significant heterogeneity was still observed. Excluding the two most heterogeneous studies [22,36] yielded a pooled OR of 0.53 with a pooled 95% CI of 0.40 to 0.70 in a combined sample of 3,946 patients (*P *< 0.00001; statistical power 99%) and moderate statistical heterogeneity and inconsistency (*Q *statistic *P *= 0.05; *I*^2 ^= 34%).

## Discussion

Patients who undergo major surgery and are managed with perioperative haemodynamic GDT are protected against hospital-acquired infectious events. Specifically, GDT decreases SSIs, pneumonia and UTIs rates, while no definite conclusions can be drawn regarding CRBSIs.

Surgical patients carry a high overall risk of hospital-acquired infections (HAIs), mainly because SSIs occur in addition to non-surgery-specific infections [47]. Despite improvements in its prevention, SSI remains one of the most frequent and expensive preventable surgical complications, occurring in up to 20% of all abdominal procedures and significantly contributing to morbidity, risk of death and financial costs [48,49]. Of note, among surgical patients, even the risk of acquiring HAIs other than SSIs is significantly higher than in those patients who are not exposed to surgery, with pneumonia and UTIs being the most frequent complications [47].

In surgical setting, several factors contribute to the likelihood of infection, depending on the complex interplay between microbial (degree of contamination and virulence), patient (immune status), and procedural characteristics (length of procedure and amount of damage to tissues). Basically, infections arise from an imbalanced relationship between bacterial load and resistance of the host patient in the context of the procedure-induced systemic inflammatory response. Surgery *per se *weakens patient responsiveness, increasing the risk of infection because of the loss of integrity of skin and mucous membranes and the impairment of microbicidal activity of the immune cells [50]. In this setting, perioperative DO_2 _plays a critical role. It is well known that adequate oxygen tissue levels promote wound healing and enhance resistance to infections [51-54]. This occurs because the oxidative burst function of neutrophils and the bacterial killing by alveolar macrophages, the primary defences against infection [55], both strictly depend on adequate oxygen tissue pressure [51,56]. Indeed, low tissue oxygen tension is among the best-established predictors of SSIs [57]. Thus, the maintenance of adequate tissue oxygen supply is a crucial step in promoting an appropriate immunological response to infection, especially in the setting of major surgery, which by itself leads to an increase in oxygen demand. Although high perioperative inspired oxygen administration represents an appealing tool, clear evidence for its beneficial effect has not yet been established. Of note, in the largest trial in which a high fraction of inspired oxygen has been tested, no influence on postoperative wound and pulmonary infections was observed [58].

In the present meta-analysis, we found that augmenting the bulk of DO_2 _by means of flow-directed therapy decreases postoperative infection rates. This finding has a pathophysiological foundation, at least theoretically. In the perioperative period, hypovolaemia and reduced cardiac output trigger musculocutaneous and splanchnic vasoconstriction, causing hypoperfusion and tissue hypoxia [1,4,59], which weaken the mucosal immunological response and disrupt the gut barrier. Gut barrier failure could lead to sepsis by means of bacteria translocation and spillover of cytokines into the bloodstream, damaging distant tissues and altering the host immunological milieu [60]. In addition, gut ischaemia-reperfusion injury markedly impairs gut-associated lymphoid tissue (GALT), further weakening extraintestinal mucosal immunity and therefore contributing to the patient's increased susceptibility to infections [60,61].

A flow-directed haemodynamic therapy aimed at optimising DO_2 _by preserving or increasing cardiac output may preserve the microbicidal function of immune cells and may protect organs particularly at risk for perioperative hypoperfusion [8], avoiding gut barrier failure and GALT impairment. Our results substantiate these concepts, demonstrating that perioperative GDT using fluid loading and/or inotropic supports to prevent tissue hypoxia could protect surgical patients against SSIs, pneumonia and UTIs.

No study has specifically assessed the effects of preserving cardiac output on SSI rates, although investigators in a recent meta-analysis concluded that supplemental hydration by itself, regardless of any specific haemodynamic target, does not affect the wound infection rate [62]. However, when the amount of perioperatively administered fluids is aimed at increasing subcutaneous oxygen tension, a greater amount of collagen in wound healing has been observed [63].

Even if the pathophysiology of postoperative pneumonia after major surgery is undoubtedly complex and multifactorial [64-66], gut failure and bacterial translocation through the lymphatics and thoracic ducts [67] and impaired respiratory mucosal immunity by a decrease in DO_2 _may have a potential pathogenetic role [68-70]. Our data seem to support this hypothesis: out of 15 studies reporting pneumonia, 13 involved abdominal surgery, which is itself a predisposing factor for gut hypoperfusion [71]. It is reasonable to argue that GDT, by ensuring adequate systemic oxygenation, can protect against ischaemia-reperfusion injury, thus decreasing the incidence of respiratory tract infections after severe surgical insults.

In surgical patients, flow-directed haemodynamic therapy aimed at optimising DO_2 _represents a useful strategy to prevent HAIs. Therefore, perioperative GDT must be strongly encouraged, particularly in high-risk surgical patients [72-74], in whom the likelihood of such complications is great.

### Limitations

The main limitations of all meta-analyses include reporting bias and the clinical and methodological heterogeneity of the included studies. To reduce reporting bias, we attempted to identify, retrieve and include all reports, grey and published [75], that met predefined inclusion criteria, and to retrieve unpublished data by contacting the authors of the included studies. No unpublished results were retrieved, and two abstracts [76,77] were identified, but they did not report the incidence of postoperative infections. Therefore, we cannot exclude the possible existence of any publication bias, and available statistical tests are not accurate enough to detect it [78].

All the studies of perioperative haemodynamic optimisation had the same starting point, fluid loading, and the same end point, achieving adequate DO_2_. However, the included studies varied in their approaches to the timing and modalities of interventions, the therapeutic targets, the monitoring tools used and the type of patients enrolled. Therefore, the clinical heterogeneity of the studies included in the present meta-analysis may be relatively high, although the statistical heterogeneity was low and the main results remained consistent across a number of subgroups and sensitivity analyses.

The paper by Mayer *et al*. [32] has been under investigation for ethical reasons because of its link to J Boldt. However, differently from other papers, their article had Institutional Review Board approval, and its publication was not retracted. Therefore, since it remained part of the scientific record at the time we searched the literature, we had no sound reason to remove this paper from our meta-analysis. However, to verify potential biases in our results, all data were reexamined without including the Mayer *et al*. [32] paper, and no differences were found.

Each type of infection (SSI, pneumonia, CRBSI and UTI) was considered separately, and a quality sensitive analysis was performed, including studies with low risk of bias (SIGN evaluation + or ++). Considering SSIs, the 14 studies reaching a low risk of bias evaluation drove the significant effect of GDT in reducing wound infections, assuring reliable and consistent evidence-based results. The four trials with a high risk of bias did not demonstrate significant results. For pneumonia, 12 of 15 studies reached a low risk of bias evaluation: The quality-sensitive analysis including only these studies confirmed the main result. The lack of outcome definition is another critical source of bias. In 10 and 6 studies, respectively, of the present meta-analysis, SSI and pneumonia were not defined. When we pooled only the studies in which the definition of SSI and pneumonia was consistent with the CDC criteria, the analyses confirmed the main results.

The pathogenesis of postoperative infections is multifactorial, involving the type of surgery, bacterial contamination and host response. Surgery itself and surgical gut manipulation are major proinflammatory stimuli [71]. All anaesthetic drugs, especially the opioids, have the potential to contribute to decreased immune response [79]. Moreover, glucose and electrolyte imbalance and hypothermia may affect immune function. We cannot state how all these conditions, comorbidities, antibiotic prophylaxis and iatrogenic interventions interact to determine infectious complications in the postoperative period.

Regarding the overall number of infectious episodes, methodological differences, including the control of covariates and, above all, the extreme variability in outcomes, may explain the presence of statistical heterogeneity and inconsistency in this result. No significant differences in CRBSIs were observed between the GDT and control groups. The very low statistical power of these analyses, owing to the low event rate, may explain this finding and does not allow us to draw any meaningful clinical conclusions, so further trials are needed.

### Research agenda

Because of the variability in methodologies and definitions of infections between studies, further prospective, randomised, controlled studies are warranted to investigate the relationship between haemodynamic GDT and infection reduction. Specifically, further studies should incorporate a rigorous protocol that includes all evidence-based approaches to reducing postoperative infections, standardises perioperative haemodynamic management and adopts a widely accepted and clinically relevant definition of 'postoperative infection' for each specific site as a primary end point. Moreover, since very recent evidence [80] supports clinical benefits and cost savings of GDT when an oesophageal Doppler monitor is used in high-risk surgical patients, further research should be done to investigate the specific role of this less invasive monitoring device in reducing postoperative infections.

## Conclusions

Infection surveillance, control and prevention systems have been developed at many centres worldwide, and numerous guidelines have been published and implemented in the effort to reduce the incidence of infections. Despite these measures, the clinical and economic burden of postoperative infections remains significant [1,2]. Our meta-analysis, within the limitations of existing data and the analytic approaches used, shows that GDT is an effective tool in reducing the incidence of infectious complications, and, more specifically, that GDT significantly decreases the rate of SSIs, pneumonia and UTIs. During surgery, GDT, by preserving or increasing cardiac output, may protect patients against severe gut ischaemia-reperfusion injury and GALT impairment, thus decreasing the incidence of postoperative infections. Hence, this strategy must be strongly encouraged. Continuing investigations into this fundamental area are warranted to better understand the link between haemodynamic optimisation and improved outcomes in surgical patients.

## Key messages

• In surgical patients, a flow-directed haemodynamic therapy aimed to optimise DO_2 _could be a useful strategy to prevent hospital-acquired infections.

• Specifically, goal-directed haemodynamic therapy significantly decreases the rate of surgical site infections, pneumonia and urinary tract infections.

• Thus, this strategy must be strongly encouraged, particularly in the setting of surgical patients at high risk for infections.

• Continuing investigations into this area are warranted to better clarify the link between haemodynamic optimisation and improved outcome in surgical patients.

## Abbreviations

ASA: American Society of Anesthesiologists; CDC: Centers for Disease Control and Prevention; CI: confidence interval; CRBSI: catheter-related bloodstream infections; DO_2_: oxygen delivery; GALT: gut-associated lymphoid tissue; GDT: goal-directed therapy; HAI: hospital-acquired infection; NNT: number needed to treat; OR: odds ratio; RCT: randomised, controlled trial; SIGN: Scottish Intercollegiate Guidelines Network; SSI: surgical site infection; UTI: urinary tract infection.

## Competing interests

The authors declare that they have no competing interests.

## Authors' contributions

LD carried out the primary study search, rechecked data and drafted and revised the manuscript. MTG extracted data, performed statistical analysis and helped to draft the manuscript. FP carried out the primary study search, rechecked data and helped to draft the manuscript. MM extracted data, tabulated quality indicators of the studies and helped to draft the manuscript. NB conceived of the study, participated in its design and coordination, extracted data and drafted and revised the manuscript. All authors read and approved the final manuscript.

## Supplementary Material

Additional file 1 **Strategies used to search the MEDLINE, Cochrane Library and EMBASE databases**. Click here for file
